# All-atom simulations and free-energy calculations of coiled-coil peptides with lipid bilayers: binding strength, structural transition, and effect on lipid dynamics

**DOI:** 10.1038/srep22299

**Published:** 2016-03-01

**Authors:** Sun Young Woo, Hwankyu Lee

**Affiliations:** 1 Department of Chemical Engineering, Dankook University, Yongin, 448-701, South Korea

## Abstract

Peptides E and K, which are synthetic coiled-coil peptides for membrane fusion, were simulated with lipid bilayers composed of lipids and cholesterols at different ratios using all-atom models. We first calculated free energies of binding from umbrella sampling simulations, showing that both E and K peptides tend to adsorb onto the bilayer surface, which occurs more strongly in the bilayer composed of smaller lipid headgroups. Then, unrestrained simulations show that K peptides more deeply insert into the bilayer with partially retaining the helical structure, while E peptides less insert and predominantly become random coils, indicating the structural transition from helices to random coils, in quantitative agreement with experiments. This is because K peptides electrostatically interact with lipid phosphates, as well as because hydrocarbons of lysines of K peptide are longer than those of glutamic acids of E peptide and thus form stronger hydrophobic interactions with lipid tails. This deeper insertion of K peptide increases the bilayer dynamics and a vacancy below the peptide, leading to the rearrangement of smaller lipids. These findings help explain the experimentally observed or proposed differences in the insertion depth, binding strength, and structural transition of E and K peptides, and support the snorkeling effect.

Membrane fusion is a process of transporting molecules between or within cells such as synaptic neurotransmission, endocytosis, and exocytosis, which is triggered by specific interactions of fusion proteins such as the formation of coiled coils^1^,^2^,^3^,^4^. For example, SNARE (soluble N-ethylmaleimide-sensitive factor attachment protein receptor) proteins^5^, which form a stable four helical coiled-coil bundle, can induce the intracellular transport, since those coiled coils on cell membranes self-assemble into a mechanically rigid bundle and thus can bring different cell membranes closer and initiate their fusion^6^,^7^. This coiled coil-induced fusion has shown great potential for drug or gene delivery because coiled coils can be attached to the drug transporter such as a liposome and modulate the entry of the transporter into cells and the release of drug molecules. To understand the mechanism of the SNARE-induced membrane fusion and develop a simple model of coiled coils for biomedical applications, synthetic mimics of SNARE proteins have been developed^8^,^9^.

The Kros group pioneered experimental studies of membrane fusion induced by synthetic coiled coils. They synthesized coiled coils “K” and “E” (respectively, (KIAALKE)_3_ and (EIAALEK)_3_; Fig. 1), which are shorter versions of SNARE proteins, and conjugated those into the liposome surface^10^,^11^. They observed the parallel-heterodimer formation of peptides E and K while still triggering membrane fusion as efficiently as SNARE proteins do. Besides this heterodimer formation, they proposed the homodimer formation of E-E and K-K, implying their aggregation on the liposome surface^12^,^13^,^14^,^15^. Recently, their compression-expression experiments with the Langmuir film showed the stronger interactions of lipid monolayers with K peptides than with E peptides^16^, which was confirmed by measuring secondary structures of peptides, indicating more helical structure for K peptides than for E peptides^17^. These results were explained by the hypothesis that lysine residues of K peptides have longer hydrocarbon chains than do glutamic-acid residues of E peptides and thus form stronger hydrophobic interactions with lipid tails, called the “snorkeling” effect, although the mechanism has not yet been understood. These imply that membrane fusion is not only induced by the coiled-coil formation of K and E peptides but also by the interactions between K peptides and membrane surfaces of their own or neighboring liposomes, which may bring neighboring liposomes closer or disorder the membrane interface. To interpret these experimental observations and assumptions, the structure of peptides and their binding strength with membranes need to be studied at nearly the atomic scale, as can be done using molecular dynamics (MD) simulations.

Bulacu and Sevink simulated E and K peptides with different types of lipid membranes using coarse-grained (CG) models, showing the homodimer formation of E and K peptides^18^. They found that E and K peptides similarly bind to unsaturated lipids, while K peptides more strongly bind to saturated lipids than do E peptides, indicating the effect of the lipid type on the peptide-membrane interaction. In contrast, Pluhackova *et al.* recently performed CG simulations and calculated free energies, showing the stronger binding of unsaturated lipids with K peptides than with E peptides^19^. They also showed the accumulation of cholesterols around K peptides and the important role of the N-terminal of K peptide for the peptide-membrane binding. These studies have revealed the conformation and interaction strength of E and K peptides with lipid bilayers, but most simulations and free energy calculations were performed using the CG model, where the helical or random-coil structure is fixed, and partial charges of atoms do not exist, which have been found to be very important for the stability of coiled coils and their interactions with lipids^20^.

In this study, we therefore perform all-atom MD simulations of E and K peptides with bilayers composed of unsaturated phospholipids and cholesterols at different ratios. First, umbrella sampling simulations are carried out to calculate free energies for binding of peptides onto the bilayer surface, showing the dependence on the lipid type. Then, we perform unrestrained simulations of peptides in lipid bilayers, showing the differences in the insertion depth, interaction strength, and structural transition of E and K peptides, which are rationalized by considering electrostatic and hydrophobic interactions between peptides and lipids. Also, the influence of peptides on the lipid dynamics and rearrangement is analyzed. We will show that these results help explain the experimentally observed or proposed differences of E and K peptides interacting with lipid membranes.

## Results

Single E or K peptides (or pairs of those peptides) were simulated with lipid bilayers composed of DOPC, DOPE, and cholesterol at different ratios. Simulated systems are named in Table 1. The initials “E” and “K” respectively indicate E and K peptides, which are followed by “PC” or “PE” that describe lipid bilayers mainly composed of DOPC (experimental condition) or DOPE, respectively. “EK”, “EE”, and “KK” indicate the dimer of E-K, E-E, and K-K, respectively. For example, “E-PE” designates a system of a single E peptide on the bilayer composed of DOPE and cholesterol at molar ratios of 3:1, while “EK-PC” indicates a system of a pair of E and K peptides on the bilayer composed of DOPC, DOPE, and cholesterol at molar ratios of 2:1:1. For umbrella sampling simulations, each window was simulated for 100 ns, leading to a total of 3200 ns for each system. To obtain more samples, three simulations were performed for each system without restraints for 350 ns.

### Free energy calculation for the peptide adsorption onto the bilayer surface

To understand the adsorption of K and E peptides onto lipid bilayers, umbrella sampling simulations were performed for a total of 12.8 μs for the systems E-PC, K-PC, E-PE, and K-PE. In Fig. 2, PMF curves were obtained from simulations of 32 windows for each system, which were calculated as a function of the distance between centers of mass (COMs) of peptide and bilayer in the bilayer normal direction, and then used to calculate free energies of binding. The lowest PMF values are found near the bilayer surface for all systems, indicating that both K and E peptides tend to bind to the bilayer. Table 2 shows that free energies are close for E-PC and K-PC, indicating almost the same adsorption strength of E and K peptides onto the bilayer surface. Free energies of E-PE and K-PE are lower than those of E-PC and K-PC, indicating that peptides bind more strongly to the bilayers mainly composed of DOPE than to those composed of DOPC.

These free energy calculations indicate that both K and E peptides tend to migrate toward the bilayer surface. In particular, those peptides more strongly bind to the DOPE/cholesterol bilayer than to the DOPC/DOPE/cholesterol bilayer, indicating the stronger adsorption onto lipid bilayers composed of smaller headgroups, presumably because smaller DOPE-amine groups do not block the attractive electrostatic interactions between lipid headgroups and peptide sidechains as much as larger DOPC-choline groups do, as also observed in simulations with antimicrobial peptides^21^.

### Insertion of peptides into lipid bilayers: conformation, helicity and structural transition

To understand the binding strength and insertion depth of peptides, the conformation and structural transition of the inserted peptides and their interactions with lipid bilayers need to be quantified, and thus we also performed unrestrained MD simulations of peptides with lipid bilayers. Figure 3 shows the initial and final snapshots from simulations. Single E or K peptides, which were initially positioned on the bilayer surface, insert into the tail region of the lipid bilayer, where hydrophobic residues of the peptide migrate toward the lipid tail region. A pair of K and E, which initially has a dimeric structure, does not insert into the bilayer, presumably because hydrophobic residues of peptides are tightly packed inside the coiled-coil dimer, which does not allow their hydrophobic interactions with lipid tails. The similar trends were also observed for other monomer and dimer systems. These configurations and insertion depth of peptides were further quantified by calculating the distances between peptide and bilayer, and mass densities. Figure 4 shows the distances between the lipid-phosphate center and the COM of each peptide in the bilayer normal direction (z-direction). Those values reach steady states at around 250 ns, indicating that simulations are equilibrated within the simulated time scale. Centers of singe peptides are close to lipid phosphates, while the coiled-coil dimer stays away from lipid phosphates, indicating the strong interactions of bilayers with single peptides but not with coiled-coil dimers. In Fig. 5, mass density profiles show that charged residues (Lys and Glu) of peptides overlap mostly with lipid headgroups, while hydrophobic residues (Ile and Leu) face toward the tail region of bilayers, again indicating strong hydrophobic interactions between peptides and bilayers, as visualized in Fig. 3. In particular, regardless of lipid types, hydrophobic residues of K peptides more broadly overlap with the lipid tail region than do those of E peptides, indicating the slightly deeper insertion of K peptides, as also observed in simulations by Pluhackova *et al.*^19^

This different binding and insertion depth of E and K peptides may be relevant to their structural differences when interacting with membranes. In fact, experiments have shown that peptides form random coils in bulk water but adopt the helical structure upon membrane binding, as well as that K peptides more strongly interact with membranes than E peptides do, indicating the structural dependence on the peptide-membrane interaction^16^,^17^. This was also supported by CG simulations that showed that when structures of K and E peptides are fixed with random coils on membranes, E peptides migrate toward water much faster than do K peptides^19^. However, the understanding of this effect requires the structural transition of peptides, and thus the secondary structure of peptides was calculated using the DSSP program^22^. In Fig. 6, secondary structures do not change much after 250 ns, again confirming that simulations are well equilibrated. Regardless of the lipid type of bilayer, K peptides retain relatively more helical structure (blue region) than E peptides do. In particular, the random-coil structure becomes more prominent for E peptides, indicating very low helicity of E peptides in lipid bilayers. Table 3 shows helicities of individual peptides for each system. Note that a broad range of helicity values are obtained from three simulations for each system, and thus those values cannot be individually compared among different systems. Also, it cannot be ruled out that helicities might be more accurately compared in the presence of more simulation data. Despite this, Table 3 clearly shows that overall helicities of K-PC and K-PE are higher than those of E-PC and E-PE, respectively. In particular, the experimental helicity of the K peptide (45-47%) is in the range of simulation values for K-PC and even very close to the average value of 46.7% from simulations, while all three helicities of E-PC are lower than 45%, showing the excellent agreement with experiments^9^,^17^. These results, combined with Figs 3, 4, 5, indicate that K peptides more deeply insert into the bilayer and retain the helical structure, while E peptides less insert and significantly lose their helicity, showing different structural transitions of K and E peptides in the bilayer, which agree quantitatively with experiments but have not yet been captured by previous CG simulations.

### Interactions between peptides and lipid bilayers: the effect of sidechain length

The above results show different structures and insertion depth of E and K peptides, which may be influenced by their electrostatic and hydrophobic interactions with lipids. For example, it has been experimentally hypothesized that hydrocarbons of Lys residues of K are longer than those of Glu residues of E, and thus K peptides may induce stronger hydrophobic interactions with membranes^16^, which was supported by simulations showing the deeper insertion of K peptides^19^, although the mechanism has not yet been understood. To resolve this, we analyzed the conformation of peptides and their interactions with bilayers.

Figure 7 shows tilt angles of peptides as a function of time. Here, the tilt angle is defined as the angle between the z-axis and the helical axis of the peptide, where the helical axis is the vector connecting from the COM of backbone atoms of the last four residues (C-terminal) to the COM of backbone atoms of the first four residues (N-terminal). Tilt angles of peptides are around 90°, indicating that peptides stay horizontally on the bilayer, which compares favorably with experiments^16^ and simulations^18^,^19^. The tilt angle of K-PC is slightly higher than others, indicating more tilted conformation. To confirm this, mass densities of peptides were calculated in terms of four residues around N- and C-terminals. Figure 7 (bottom) shows that N-terminal of K peptide is more deeply inserted than C-terminal, while both terminals of E peptide are almost completely overlap, indicating the deeper insertion of N-terminal of K peptide, as observed in recent CG simulations with amino-acid mutation studies^19^.

**Figure 1:**
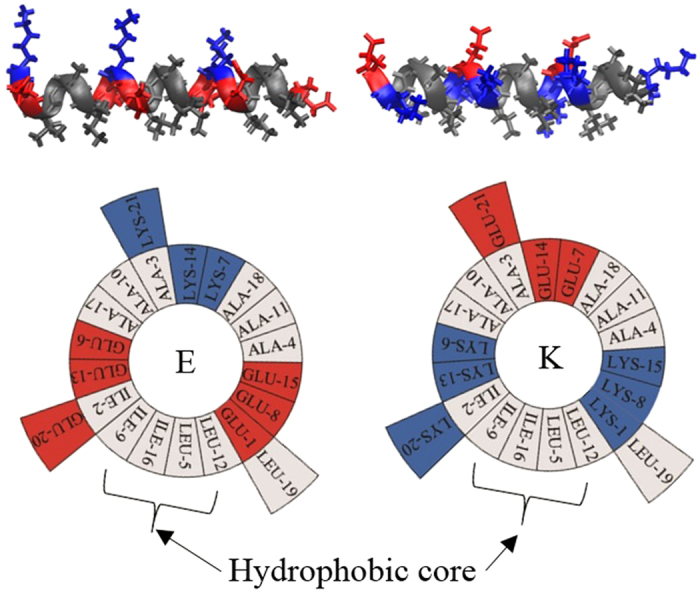
Structures and helical wheel diagrams for the peptides E (left) and K (right). Peptides are represented as ribbons. For the wheel diagrams, amino acid sequences are plotted clockwise. Hydrophobic, anionic, and cationic residues are colored in gray, red and blue, respectively. The structure images were created with Visual Molecular Dynamics^44^.

To understand the stronger membrane interaction with K peptide than with E peptide, radial distribution functions (RDFs) between peptides and lipids were analyzed. Note that E and K peptides have the same hydrophobic-core sequence but different charged residues adjacent to the hydrophobic core (Fig. 1), implying that those charged residues may modulate the interactions between peptides and membranes. Thus, we calculated RDFs of charged terminal groups of peptide’s Lys and Glu with respect to lipid choline and phosphate groups, and RDFs of hydrocarbons of Lys and Glu with respect to lipid tail groups. Figure 8 shows that charged terminal groups of Glu and Lys respectively interact with cationic cholines (or amines) and anionic phosphates of lipids, as expected because of their electrostatic interactions. Interestingly, RDFs between hydrocarbons and lipid tails show much higher peaks for K peptides than for E peptides, indicating much stronger hydrophobic interactions between K peptides and lipid tails.

These results, combined with free energy calculation, show that both E and K peptides tend to migrate toward the bilayer surface and more strongly bind to the bilayer mainly composed of DOPE than to the bilayer composed of DOPC, showing the effect of the lipid headgroup type. However, after inserting into the bilayer, the interaction strengths of E and K peptides differ. K peptides more deeply insert into the bilayer and partially retain the helical structure, while E peptides less insert and predominantly have the random-coil structure, as observed in experiments^16^,^17^. This is because charged sidechains of E and K peptides respectively interact with lipid choline and phosphate groups, implying that since the phosphate group is closer to the bilayer center than is the choline group, K peptides can more easily migrate toward the lipid tail region than do E peptides. In particular, K peptides form stronger hydrophobic interactions with lipid tails due to their longer hydrocarbon chains, which explains the deeper insertion of K peptide and its retention of stable helical structure, as well as supports the experimentally proposed snorkeling effect.

### Effects of peptides on the bilayer dynamics and arrangement

**Figure 2:**
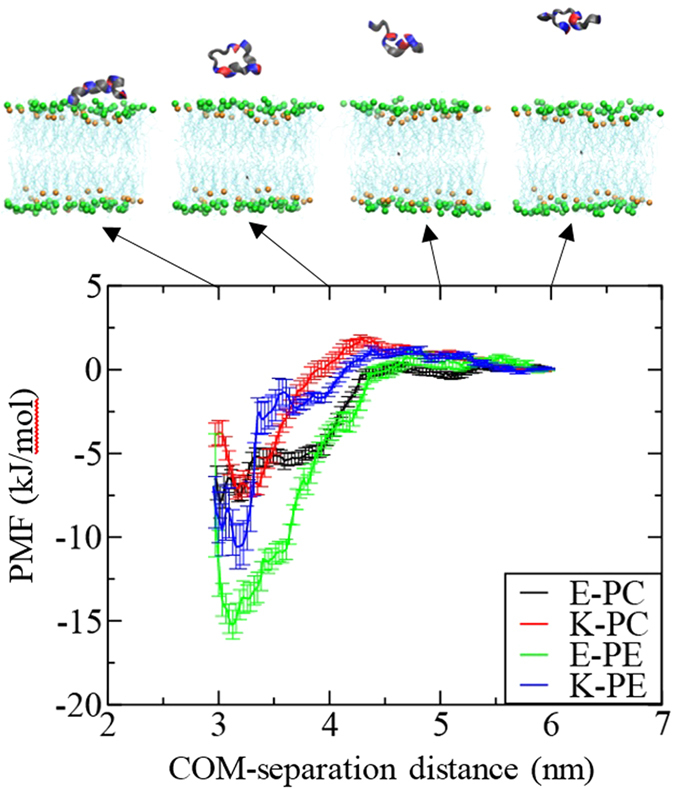
Snapshots of umbrella sampling simulations (top) and potentials of mean forces (PMF) for peptides binding to the bilayer surface (bottom) as a function of the reaction coordinate, which is the z-axial distance of 6–2.8 nm between peptide and bilayer centers. In the snapshots, green and orange dots respectively represent phosphorous atoms of lipid head groups and oxygen atoms of cholesterols, while light-blue lines indicate lipid tail groups. Water and ions are omitted for clarity.

**Figure 3:**
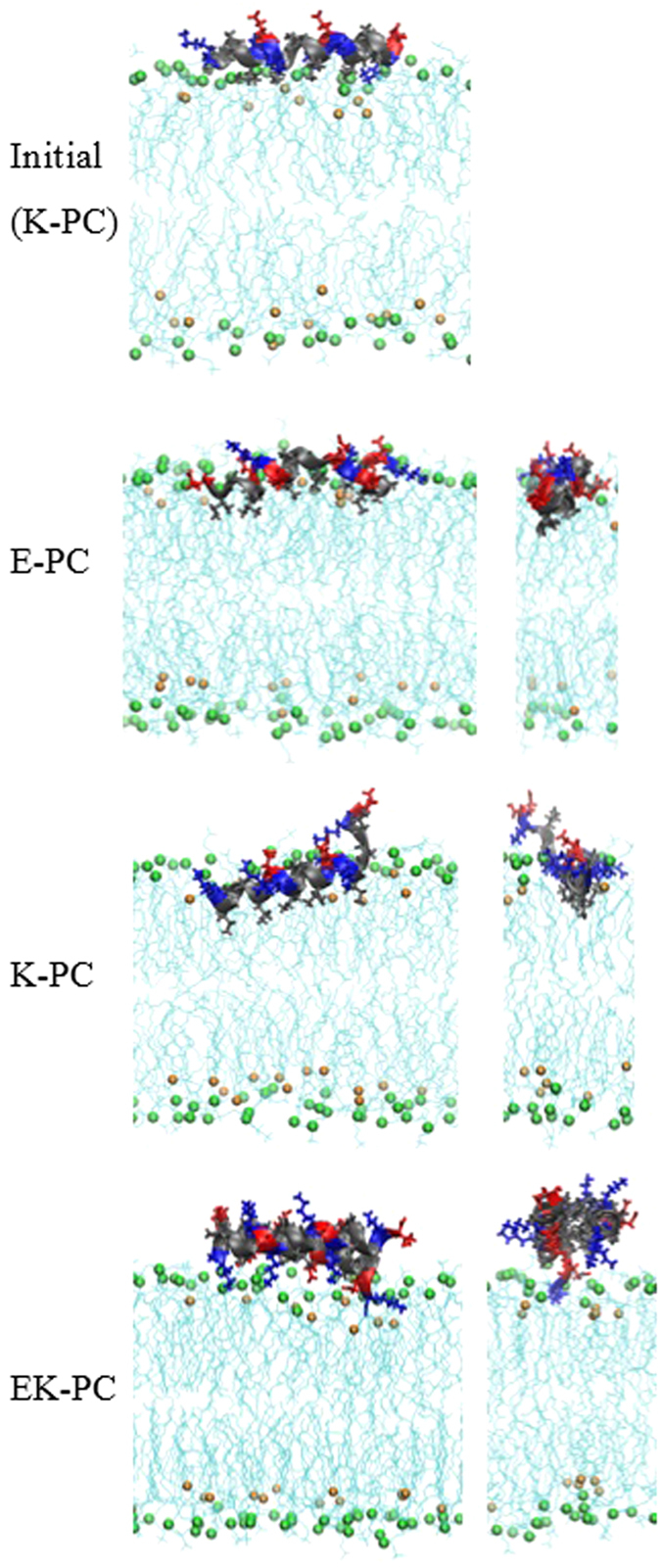
Snapshots at the beginning (0 ns; top) and the end (350 ns; rows 2–4) of simulations. Initial configuration is shown only for K-PC, but this initial configuration is also applied for all other systems. Final configurations are represented in terms of the longest and shortest axes. Peptide and bilayer colors are the same as those in Figs 1 and 2. The explicit water and counterions are omitted for clarity.

**Figure 4:**
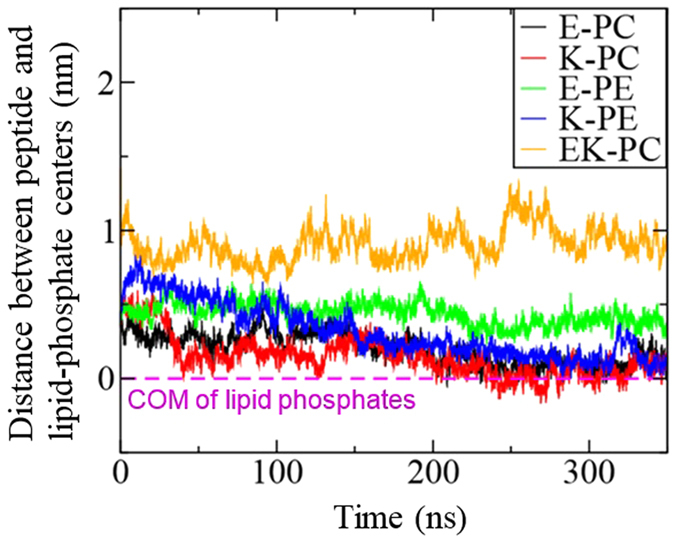
Distances between centers of mass of the peptide and the lipid phosphate in the bilayer normal direction (z-direction) as a function of time.

**Figure 5:**
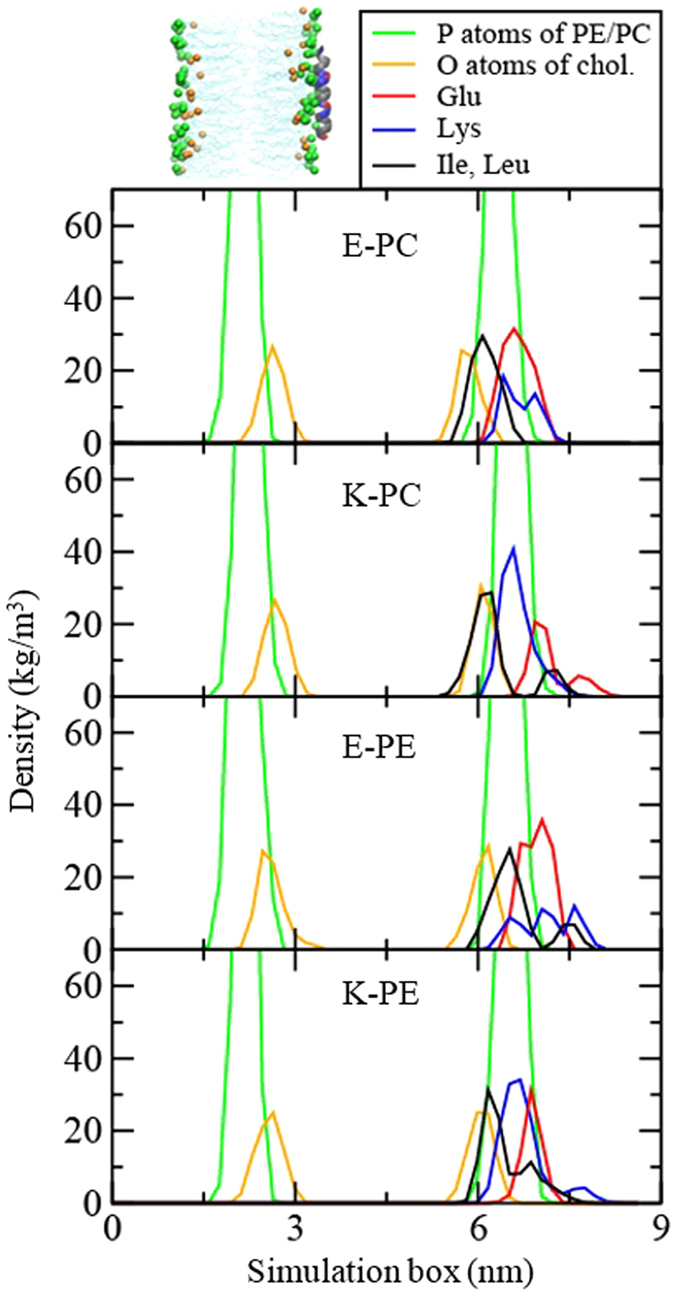
Mass density profiles of peptides, lipids, and cholesterols.

**Figure 6:**
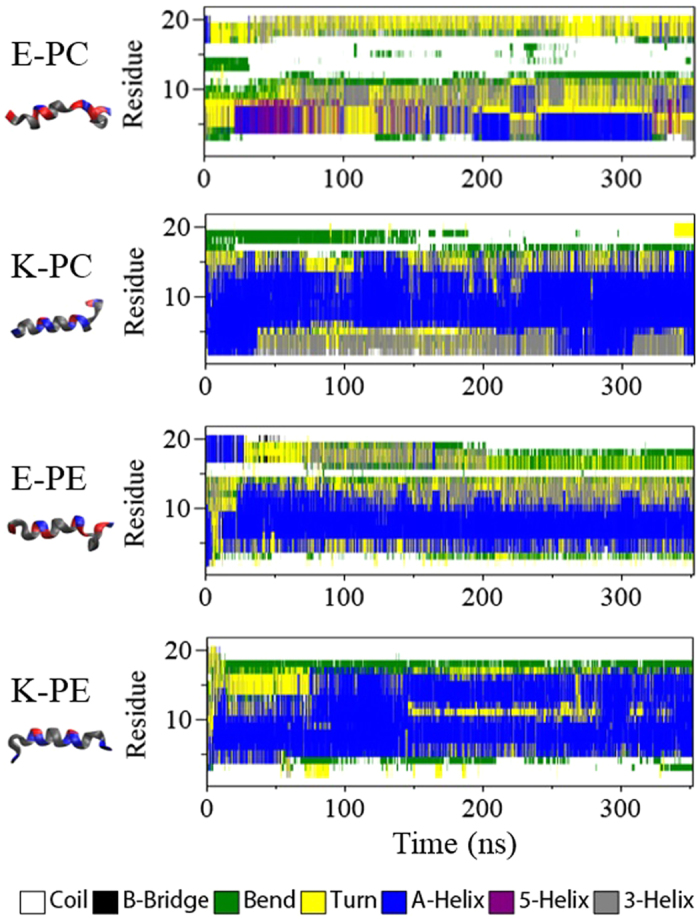
Secondary structure profiles of peptides as a function of time.

**Figure 7:**
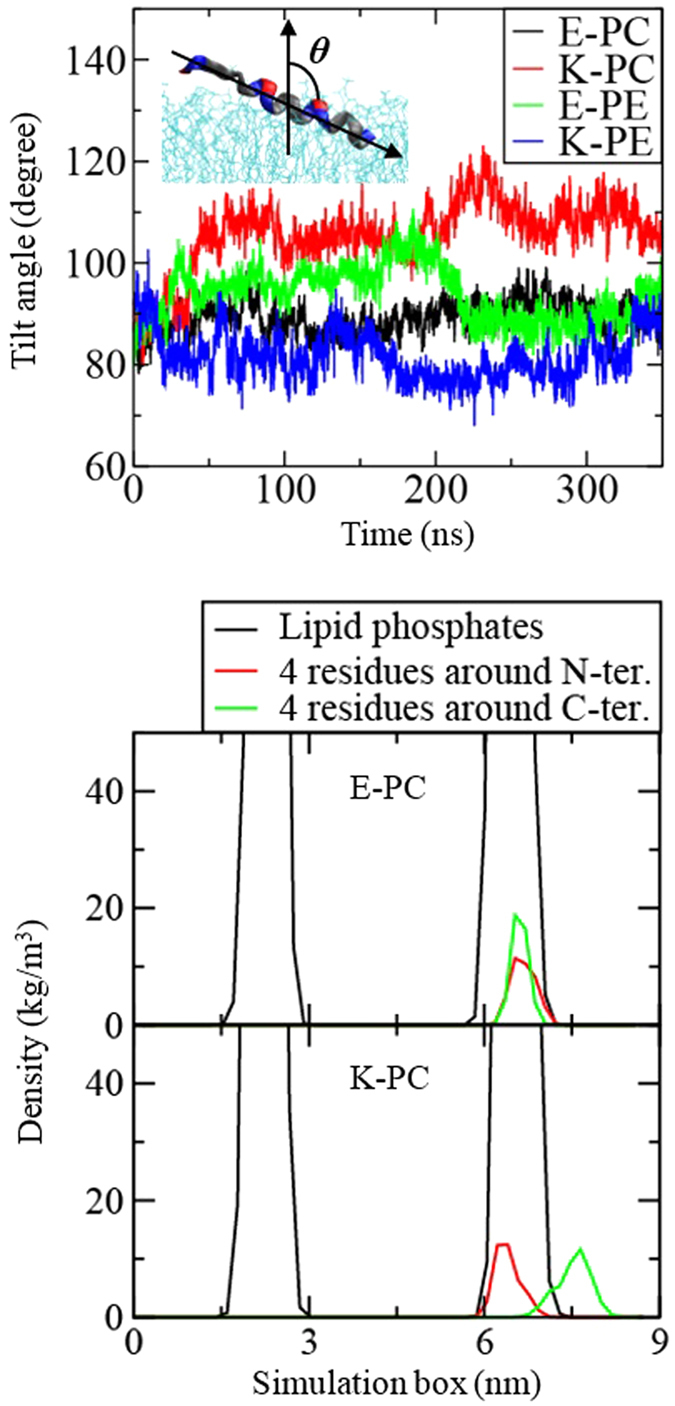
Tilt angles of peptides as a function of time (top) and mass density profiles of lipid phosphates and four residues around N- and C-terminals (bottom).

**Figure 8:**
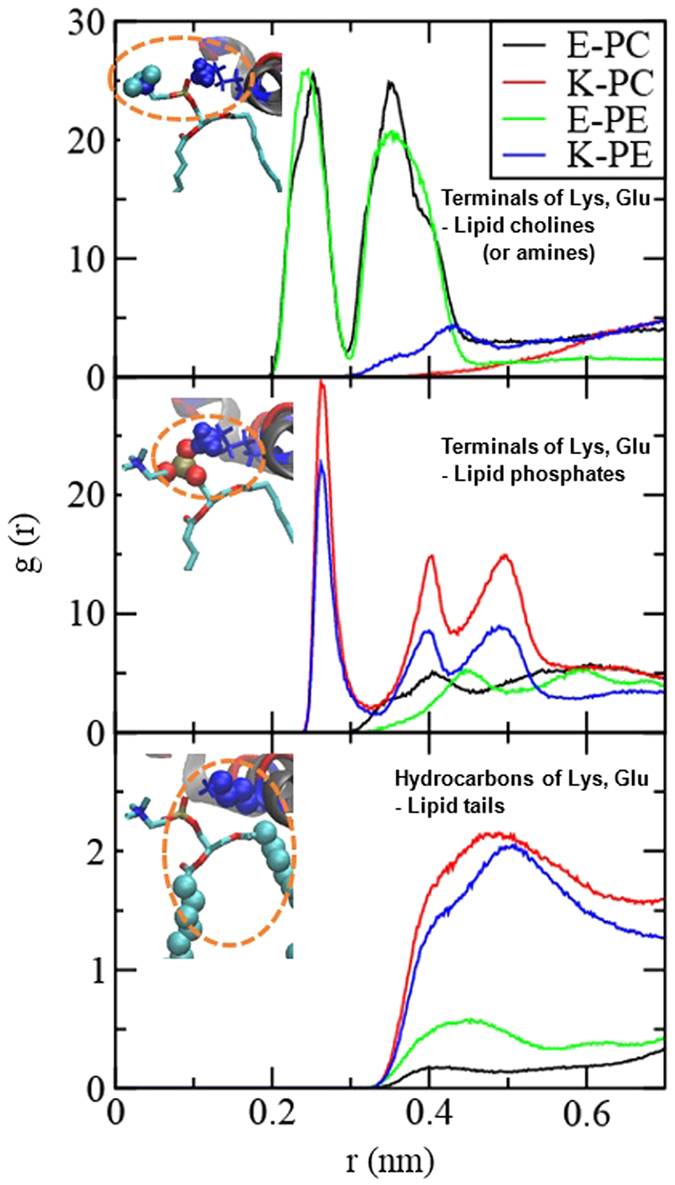
Radial distribution functions (RDFs) of charged terminal groups of Lys and Glu with respect to the lipid choline (or amine) group (top) and the lipid phosphate group (middle) and RDFs of hydrocarbons of Lys and Glu with respect to the lipid tail group (bottom).

**Figure 9:**
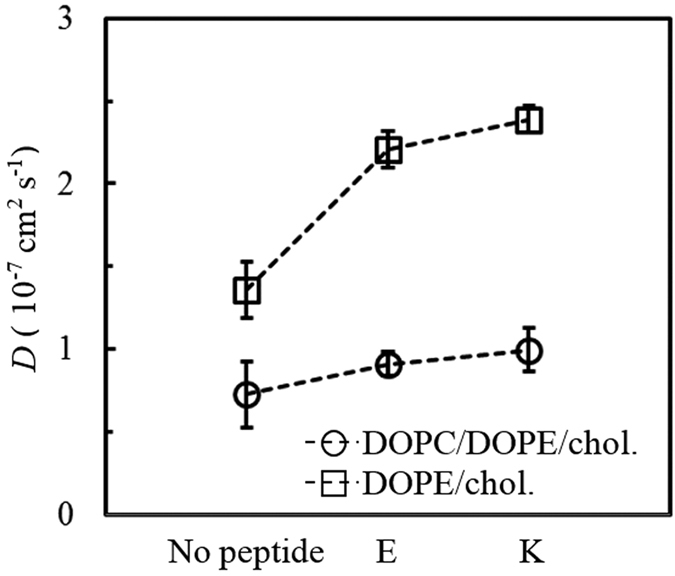
Lateral diffusion coefficients (*D*) of lipids.

Since the membrane-interaction strengths of E and K peptides differ, their effects on the membrane dynamics and properties should also differ. To check this, lateral diffusion coefficients of lipids were calculated from the slopes of the mean-square displacements (MSD) in the xy-plane (the direction perpendicular to the bilayer normal). Figure 9 shows that the presence of peptides increases the lateral diffusivity of the bilayer, as expected since the inserted peptides can disorder lipids. In particular, K peptides induce the higher diffusivities than do E peptides, indicating that K peptides more effectively disorder lipids, which confirms the stronger interaction of K peptides. Also, this effect occurs more significantly in the DOPE/cholesterol bilayer than in the DOPC/DOPE/cholesterol bilayer, also confirming that peptides more strongly bind to the bilayer with smaller lipid headgroups and thus disorder the bilayer composed of DOPE more significantly than the bilayer mainly composed of DOPC, consistent with free energy calculation in Fig. 2 and Table 2.

**Figure 10:**
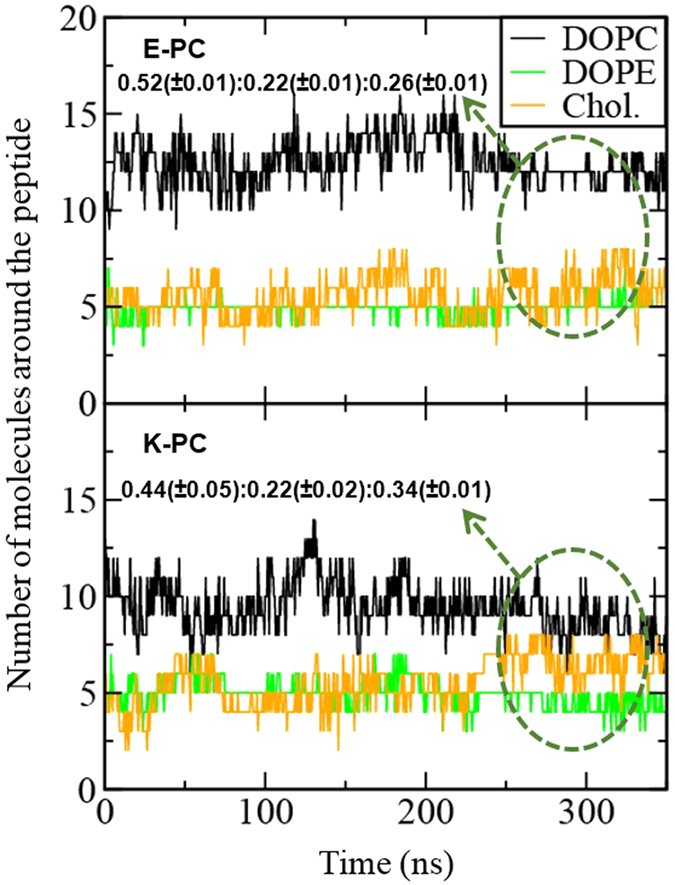
Numbers of DOPCs, DOPEs, and cholesterols within a distance of 0.8 nm from the peptide as a function of time. Average molar ratios (DOPC:DOPE:cholesterol) are calculated.

**Figure 11:**
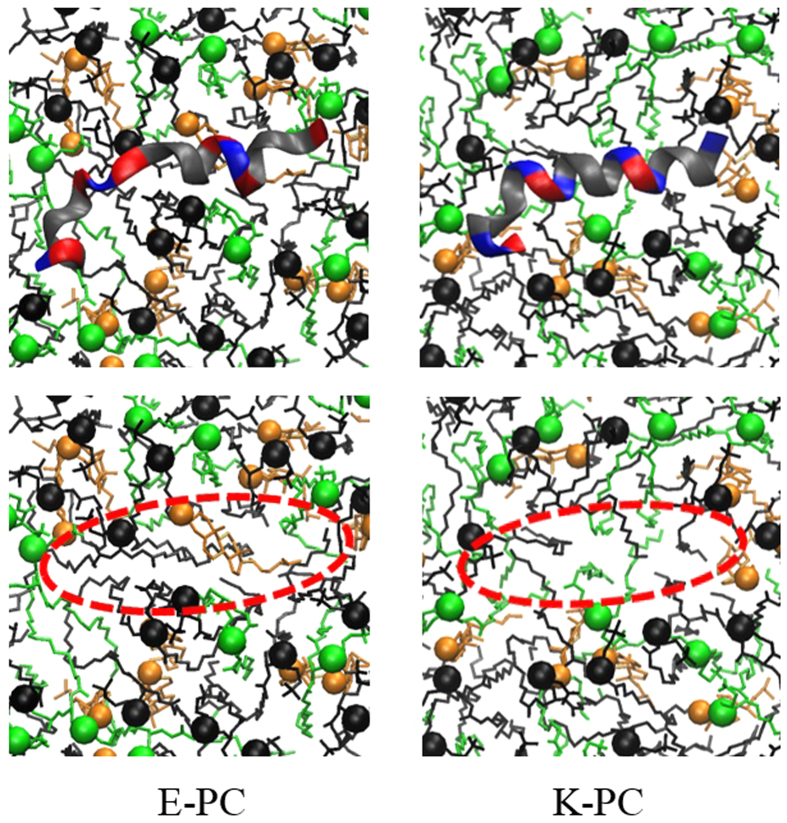
Snapshots of the top view at the end of simulations for E-PC (left) and K-PC (right). For each system, the peptide is visually removed to clearly see the lipid arrangement (bottom). Black, green, and orange colors respectively represent DOPC, DOPE and cholesterols with highlights of lipid phosphorus and cholesterol O atoms as colored dots.

This increased dynamics of the bilayer may also influence the arrangement of lipids and cholesterols around the peptide, and hence the numbers of DOPC, DOPE, and cholesterol within a distance of 0.8 nm from the peptide were computed. In Fig. 10, E-PC shows that the numbers of those molecules do not change much, leading to the average molar ratio of 0.52:0.22:0.26, whereas K-PC shows that the number of DOPC significantly decreases, leading to the molar ratio of 0.44:0.22:0.34. This indicates fewer DOPC molecules around the K peptide than around the E peptide. The similar trend was also observed in previous CG simulations that showed more cholesterols around K peptide^19^, although we here observe a decrease of DOPC rather than an increase of cholesterol. A comparison of lipid arrangements around E and K peptides is visualized in Fig. 11. These images show that there is a larger vacancy with fewer DOPCs below the K peptide, implying that the deeper insertion of the K peptide does not allow enough space for lipids and surfactants below the peptide, especially for DOPC that has the larger headgroup. These results indicate that since K peptides more deeply insert into the bilayer than do E peptides, K peptides more effectively increase lateral dynamics of lipids and tend to have smaller lipids and surfactants around the peptide.

## Discussion

We performed all-atom MD simulations of synthetic peptides E and K with lipid bilayers composed of DOPC, DOPE, and cholesterol at different molar ratios. We first performed umbrella sampling simulations and calculated free energies for binding of peptides onto to the bilayer surface, showing that both E and K peptides tend to migrate toward the bilayer surface, which occurs more significantly in the DOPE/cholesterol bilayer than in the DOPC/DOPE/cholesterol bilayer. This indicates that peptides more strongly bind to the lipid bilayer with smaller headgroups, since smaller headgroups do not block the electrostatic interactions between peptides and lipids as much as larger ones do. Then, we performed unrestrained MD simulations of peptides initially partially inserted into the bilayer, showing that pairs of E and K peptides do not insert into the bilayer because their hydrophobic residues are tightly packed inside the coiled-coil dimer, while single E or K peptides insert into the bilayer due to their hydrophobic interactions with lipid tails. K peptides more deeply insert into the bilayer than E peptides and partially retain the helical structure, while E peptides less insert and predominantly become random coils, leading to the structural transition from helices to random coils, in quantitative agreement with experiments. This is because Lys residues of K peptides electrostatically interact with lipid phosphates, which are close to the bilayer center, as well as because longer hydrocarbons of Lys residues of K peptide can form stronger hydrophobic interactions with lipid tails than do shorter hydrocarbons of Glu residues of E peptide, leading to the deeper insertion of K peptide. This deeper insertion of K peptide induces the faster lateral dynamics of bilayers and a larger vacancy below the peptide, leading to the rearrangement of smaller lipids around the peptide. These simulation findings help explain the experimentally observed or proposed differences in the insertion depth, interaction strength, and structural transition of E and K peptides, and also support the snorkeling effect.

## Methods

### Unrestrained simulations

All simulations and analyses were performed using the GROMACS4.6.7 simulation package^23^,^24^,^25^ with the OPLS all-atom force field (FF) and the TIP4P water model^26^,^27^. The coordinates of E and K peptides were downloaded from the Protein Data Bank (PDB code: 1U0I)^28^. The N- and C-termini were respectively unprotonated and protonated to make them electrostatically neutral, matching the terminal charge state of the experimental peptides^16^. Potential parameters for dioleoylglycerophosphocholine (DOPC) and dioleoylglycerophosphoethanolamine (DOPE) lipids were taken from the Berger lipid FF^29^ modified by Tieleman *et al.*^30^, which can be compatibly used with the OPLS protein FF and has successfully predicted the experimentally observed areas per lipid and dynamics of DOPC and other phospholipid bilayers^31^,^32^,^33^. For cholesterol, we used potential parameters developed by Holtje *et al.*^34^, which was converted to be used with the OPLS FF by Hub *et al.*^35^

Bilayer systems were generated with the mixture of DOPC, DOPE, and cholesterol at different molar ratios of either 2:1:1, close to the experimental condition^12^,^15^,^16^, or 0:3:1 (Table 1). A single K or E peptide (or a pair of peptides) was positioned above the equilibrated bilayer surface with a distance of 0.4 nm between the peptide and lipid-phosphate centers. The final simulation systems consist of a K or E peptide (or a pair of peptides), 128 lipids or surfactants (64 lipids/leaflet), ~4500 water molecules, and 3 counterions (either Na^+^ or Cl^−^) in a periodic box of size 5.4 × 5.5 × 9 nm^3^. A real space cutoff of 14 Å was applied for Lennard-Jones potential and electrostatic forces with the inclusion of particle mesh Ewald summation for long-range electrostatics^36^. A temperature of 298 K and a pressure of 1 bar were maintained by applying the velocity-rescale thermostat^37^ and the Berendsen barostat^38^ in an NP_xy_P_z_T ensemble (with semi-isotropic pressure coupling). The LINCS algorithm was used to constrain the bond lengths^39^,^40^. To obtain more samples, three simulations were performed for each peptide-bilayer system. Simulations were carried out for 350 ns with a time step of 2 fs on computational facilities supported by the DGIST Supercomputing & Bigdata Center with the allocation of the superc mputing time and technical supp rt, and the last-100 ns trajectories were used for analyses.

### Umbrella sampling simulations

Potentials of mean force (PMF) were calculated using the umbrella sampling algorithm^41^. Starting with an initial position of a peptide with a distance of 6 nm above the lipid-bilayer center, the peptide was pulled toward the bilayer surface along the direction of the bilayer normal (z-axis) with a force constant of 1000 kJ mol^−1 ^nm^−2^ (Fig. 2, top), which yields a total of 32 windows with a window spacing of 0.1 nm in the distance of 6–2.8 nm from the bilayer center. These 32 windows were equilibrated for 100 ps and then used as starting configurations for umbrella sampling simulations. Each window was simulated for 100 ns, leading to a total of 3200 ns for 32 windows of each peptide-bilayer system. The last-80 ns trajectories were used to unbias umbrella samplings using the weighted histogram analysis method (WHAM)^42^. Errors were estimated from the bootstrapping analysis, called the Bayesian bootstrapping of complete histograms, where random weights are assigned to all histograms within each bootstrap^43^.

## Additional Information

**How to cite this article**: Woo, S. Y. and Lee, H. All-atom simulations and free-energy calculations of coiled-coil peptides with lipid bilayers: binding strength, structural transition, and effect on lipid dynamics. *Sci. Rep.* **6**, 22299; doi: 10.1038/srep22299 (2016).

## Figures and Tables

**Table 1:** List of simulations.

		No. of molecules					No. of simulations (or windows for PMF)	Simulation time (ns)
		Peptide		Lipid bilayer				
		E	K	DOPC	DOPE	Cholesterol		
Umbrella	E-PC	1		64	32	32	32	3200
sampling	K-PC		1	64	32	32	32	3200
(PMF)	E-PE	1			96	32	32	3200
	K-PE		1		96	32	32	3200
Unrestrained	E-PC	1		64	32	32	3	350
simulation	K-PC		1	64	32	32	3	350
	E-PE	1			96	32	3	350
	K-PE		1		96	32	3	350
	EK-PC	1	1	64	32	32	1	350
	EE-PC	2		64	32	32	1	350
	KK-PC		2	64	32	32	1	350

**Table 2:** Free energies for binding of peptides to the bilayer surface.

	Free energy (kJ mol^−1^)
E-PC	−7.9 ± 0.9
K-PC	−7.1 ± 0.6
E-PE	−15.6 ± 0.9
K-PE	−10.6 ± 1.3

**Table 3:** Helicities (%) of individual K and E peptides in lipid bilayers. Since three simulations were performed for each system, there are three helicity values.

	Helicity (%)	
	This work	Experiment^9^,^17^
E-PC	30 ± 1	38
	19 ± 2	
	6 ± 3	
K-PC	64 ± 1	45–47
	50 ± 1	
	26 ± 1	
E-PE	42 ± 1	–
	33 ± 2	
	14 ± 3	
K-PE	52 ± 2	–
	45 ± 2	
	35 ± 1	
